# Metabolomic Characterisation of Discriminatory Metabolites Involved in Halo Blight Disease in Oat Cultivars Caused by *Pseudomonas syringae* pv. *coronafaciens*

**DOI:** 10.3390/metabo12030248

**Published:** 2022-03-16

**Authors:** Chanel J. Pretorius, Paul A. Steenkamp, Fidele Tugizimana, Lizelle A. Piater, Ian A. Dubery

**Affiliations:** Research Centre for Plant Metabolomics, Department of Biochemistry, University of Johannesburg, Auckland Park, Johannesburg 2006, South Africa; 201423600@student.uj.ac.za (C.J.P.); psteenkamp@uj.ac.za (P.A.S.); ftugizimana@uj.ac.za (F.T.); lpiater@uj.ac.za (L.A.P.)

**Keywords:** *Avena sativa*, LC–MS, metabolomics, multivariate data analysis, oat, *Pseudomonas syringae* pv. *coronafaciens*, secondary metabolites

## Abstract

The metabolome is the underlying biochemical layer of the phenotype and offers a functional readout of the cellular mechanisms involved in a biological system. Since metabolites are considered end-products of regulatory processes at a cellular level, their levels are considered the definitive response of the biological system to genetic or environmental variations. The metabolome thus serves as a metabolic fingerprint of the biochemical events that occur in a biological system under specific conditions. In this study, an untargeted metabolomics approach was applied to elucidate biochemical processes implicated in oat plant responses to *Pseudomonas syringae* pv. *coronafaciens* (*Ps-c*) infection, and to identify signatory markers related to defence responses and disease resistance against halo blight. Metabolic changes in two oat cultivars (“Dunnart” and “SWK001”) responding to *Ps-c*, were examined at the three-leaf growth stage and metabolome changes monitored over a four-day post-inoculation period. Hydromethanolic extracts were analysed using an ultra-high-performance liquid chromatography (UHPLC) system coupled to a high-definition mass spectrometer (MS) analytical platform. The acquired multi-dimensional data were processed using multivariate statistical analysis and chemometric modelling. The validated chemometric models indicated time- and cultivar-related metabolic changes, defining the host response to the bacterial inoculation. Further multivariate analyses of the data were performed to profile differential signatory markers, putatively associated with the type of launched defence response. These included amino acids, phenolics, phenolic amides, fatty acids, flavonoids, alkaloids, terpenoids, lipids, saponins and plant hormones. Based on the results, metabolic alterations involved in oat defence responses to *Ps-c* were elucidated and key signatory metabolic markers defining the defence metabolome were identified. The study thus contributes toward a more holistic understanding of the oat metabolism under biotic stress.

## 1. Introduction

Due to their sessile nature, plants have developed a range of defence mechanisms against biotic and abiotic stresses. One such mechanism includes the initiation of adaptive responses, such as stress recognition, signal transduction, activation of several stress-related genes accompanied by the production and activation of secondary metabolites or specialised phytochemicals [1]. A range of metabolites act as constitutive defence compounds prior to the pathogenic attack (phytoanticipins), i.e., these preformed compounds occur in healthy plants. Other defence compounds are inducible and released upon pathogen infection and are known as phytoalexins [2,3,4]. Disease resistance can be viewed as a continuum of responses ranging from highly resistant (no disease symptoms) to tolerant (some disease symptoms) to highly susceptible (significant disease symptoms). Changes in plant metabolism are key in understanding and analysing the result of attempted infections. Metabolomic analyses of healthy and newly infected plants, could thus provide invaluable information on changes in signalling or output pathways [5,6]. Plant metabolism involves the regulation of different metabolic processes that enable plants to withstand environmental threats. Identified primary metabolites (like carbohydrates, amino and organic acids) have been greatly studied to elucidate to what extent plant metabolism is altered to adapt to an ever-changing environment [7,8]. Secondary metabolites, on the other hand, also include chemically diverse phytoalexin compounds, synthesised to counterattack various pathogenic organisms, and key components in understanding plant response and defence mechanisms. Important also, are phytohormones (e.g., auxins, cytokinins, gibberellins, jasmonates or jasmonic acid (JA), abscisic acid (ABA) and salicylic acid (SA), central for plant signalling in response to stimuli from the abiotic/biotic environment (Supplementary Figure S1) [9,10,11].

Oat (*Avena sativa* L.) crops rank among the top six most important cereals in the world, important for human consumption, livestock feed, fodder, forage, hay and silage. Due to its hardiness and ability to grow and resist change under adverse environmental conditions, oat crops are also considered a superior cereal crop [12]. Many of these benefits are due to the numerous bioactive phytochemicals present in oat plants, such as phenolic acids, flavonoids, phytosterols and carotenoids, to name a few. Additionally, this cereal also produces unique phytochemicals known as avenanthramides, avenacosides and avenacins [13,14]. Like all plants, oat crops are often exposed to a range of pathogens that can lead to severe disease and great crop losses. *Pseudomonas syringae* pv. *coronafaciens (Ps-c)* is the common cause of halo blight disease in oat crops [15], resulting in yield- and economic losses [16,17,18,19]. It has been known to cause a hypersensitive response (HR) on oat leaves that leads to necrotic spots characterised by small, oval-shaped, water-soaked spots found on leaves in early onset of the disease and soon change to reddish-brown, oval-shaped lesions with a light centre and a characteristic yellow halo surrounding the lesions. In severe cases of infection, young leaves become curved and chlorotic without the presence of necrotic spots or broad yellow halos [20]. Environmental conditions can affect disease severity, development and spread of halo blight disease as *Ps-c* favours moist conditions and optimally spreads through rain, wind and insects [21,22].

The general fundamental concepts of plant innate immunity have been well established and involve the recognition of pathogen-associated molecular pattern (PAMP) molecules by cell surface located receptors as well as intracellular recognition of pathogen effector proteins by resistance (R) proteins from the plant host [23]. This implies a strong genetic basis for resistance or susceptibility against a specific disease, that is ultimately reflected in the metabolomic phenotype [24]. Here an untargeted liquid chromatography coupled to mass spectrometry LC–MS-based metabolomics approach was used to detect underlying metabolic alterations and to identify potential metabolic markers that contribute to oat defence in response to inoculation with *Ps-c*. While some oat cultivars exhibit a higher level of disease resistance, the molecular mechanisms underlying these interactions are still poorly understood [25]. By analysing the cellular and molecular responses between the plant and pathogen, sustainable means of combating disease could be developed and be particularly useful in breeding programs by identifying metabolic markers associated with resistance or susceptibility [26].

## 2. Results

### 2.1. Disease Severity and Symptom Development of Halo Blight in Oat Cultivars 

**Figure 1:**
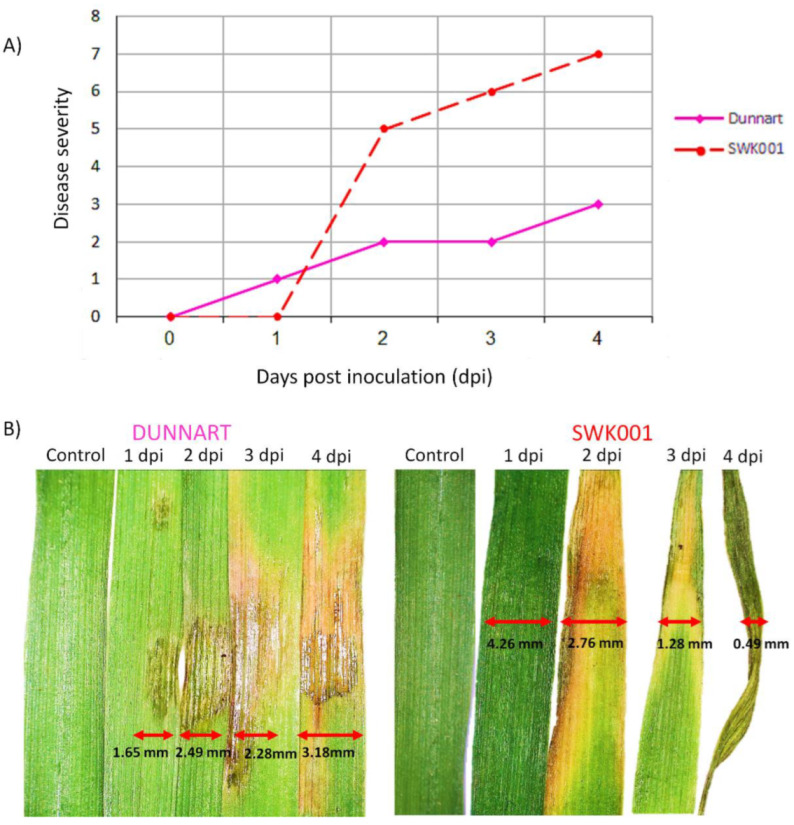
Disease severity rating and typical symptom development of oat cultivars responding to *Pseudomonas syringae* pv. *coronafaciens* infection over a period of 4 days post-inoculation (d.p.i.). (**A**) Disease severity scores for phenotypical symptom development in “Dunnart” (solid pink line) and “SWK001” (dashed red line) cultivars ranging from 0 = leaves are free of any visual symptoms to 8 = very severe wilting occurs (50% or more of leaves are yellow and wilted). (**B**) Following inoculation, the development of lesions on the “Dunnart” leaves were observed over time. Lesions started appearing from 1 d.p.i. and spread progressively over time. At 4 d.p.i. the lesions appear to cover a larger section of the leaf surface. In the “SWK001” cultivar severe symptoms can be seen as the leaves start yellowing from the tips at 2 d.p.i. and become more severe over time. At 4 d.p.i. the leaves start dying and appear shrivelled.

The disease severity and development of symptoms in the “Dunnart” and “SWK001” (Figure 1A,B) show cultivar-related differential interactions with *Ps-c*. Halo blight disease was evaluated by visual observation using a 0–8 scale, where 0 = no disease symptoms, 1–3 = slight disease symptoms, 3–6 = moderate disease symptoms and 6–8 = severe symptoms of yellowing and wilting (Supplementary Figure S2 and Figure 1A) [27]. “Dunnart” showed typical halo blight symptoms such as the small water-soaked spots on the surfaces of the leaves. As the symptoms developed a characteristic yellow halo appeared around the spots (Figure 1B). This halo is due to the action of a toxin produced by the bacteria and is a diagnostic symptom of the disease [28]. In severe infections, symptoms commonly include the leaves and upper parts of the plant turning yellow (chlorotic) and shrivelled [29] as can be seen with the “SWK001” cultivar (Figure 1B).

The phenotypic symptoms and observations suggest that “Dunnart” exhibits a stronger defence response compared to the “SWK001” cultivar, with HR-like lesions and yellow halo development around the affected tissue (Supplementary Figure S3 and Figure 1B). In contrast, the “SWK001” cultivar showed minimal/no resistance against *Ps-c* as this cultivar developed severe symptoms (Figure 1B). The development of halo blight and its severity in oat plants differ depending on the interaction between the pathogen and plant, as well as the environmental conditions [12]. It is also important to consider the specific variety or cultivar, as significant variation may occur within cultivars from the same species with regard to their susceptibility or resistance to specific diseases [30]. In this study, the two oat cultivars [24] were infected under a controlled environment and symptomatic differences thus illustrate only gene-directed, cultivar-related responses to the *Ps-c* inoculation. Plants have developed sophisticated and inherently complex multi-layered surveillance—and defence mechanisms as part of innate immunity to survive changing environments and pathogenic threats [31]. Therefore, characterising the metabolic phenotypes associated with oat defence response to *Ps-c* infection would allow for greater insight into the cellular and metabolic pathways involved in the plant-pathogen interaction [32].

### 2.2. Liquid Chromatography-Mass Spectrometry-Based Analyses of Oat Response to *Ps-c*

**Figure 2:**
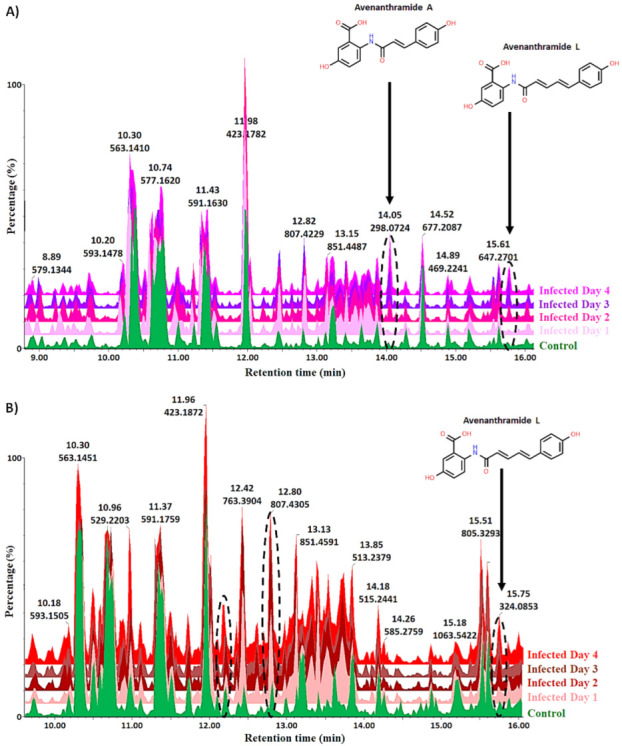
Ultra-high-performance liquid chromatography (UHPLC) coupled to mass spectrometric (MS) detection in negative electrospray ionisation (ESI) mode. The figure compares base peak intensity (BPI) MS chromatograms of extracts at the seedling stage compared to the non-treated control (uninfected plants) for (**A**) “Dunnart” and (**B**) “SWK001”. These are reverse phase chromatographic separations based on the polarity of the respective compounds. The dashed oval structures highlight certain unique variants that show how the phytochemical profiles of the infected cultivars changed over time. As illustrated, avenanthramide phytoalexins were shown to be absent in the control(s) and increase in abundance over time and, although only avenanthramide A and L are shown, four different avenanthramides (A, B, C and L) were detected.

Hydromethanolic leaf extracts of the infected, non-infected (vehicle control) and negative control oat plants were analysed on a reversed-phase UHPLC–qTOF–MS system (ultra-high-performance liquid chromatography coupled to quadrupole time-of-flight mass spectrometry). Based on initial optimisation experiments, electrospray ionisation (ESI) in negative mode showed better ionisation efficiency. The acquired ESI(−) data were thus further analysed and is illustrated throughout. To elucidate and identify as many statistically significant metabolites as possible, an untargeted approach was used. Chromatographic analysis, which separates components based on their polarity resulting in high-resolution separation and analysis of sample constituents, provided essential information on the innate biochemical diversity of plants and the multi-dimensionality of the extracted metabolomics data [33,34]. The coupling of chromatography to mass spectrometry provided a highly sensitive analytical platform that permitted the simultaneous detection of a range of metabolites to provide a more holistic understanding of the metabolic composition of the biological samples [8]. Figure 2 illustrates the base peak intensity (BPI) chromatograms with distinct peak populations showing the differences and similarities between the infected and non-treated control groups for both cultivars. The examples illustrated by the chromatographic separation as changes between the infected and non-treated control groups include the increasing abundance of avenanthramide A and L in the infected “Dunnart” cultivar compared to the control group from 1–4 d.p.i. (Figure 2A) and increasing abundance of avenanthramide L in the infected “SWK001” cultivar from 1–4 d.p.i. compared to the control (Figure 2B). To gain deeper insights into the underlying biochemical changes related to the oat response upon treatment with *Ps-c*, the complex, multi-dimensional data sets were further analysed using chemometrics and multivariate data analysis. 

### 2.3. Chemometrics for the Analyses of Halo Blight-Induced Metabolic Changes in Oat Cultivars

**Figure 3:**
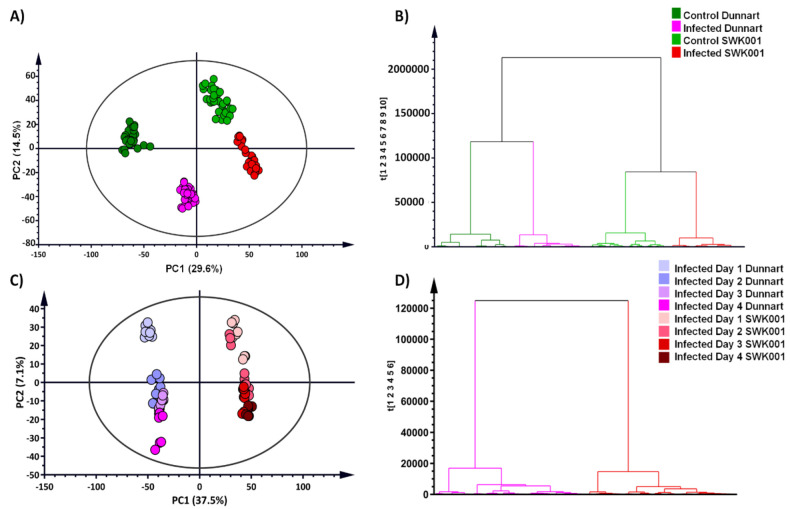
Principal component analysis (PCA) of the two infected oat cultivars with the corresponding hierarchical cluster analysis (HCA) dendrograms. PCA scores plots indicate the clustering and general grouping among the infected and control groups for “Dunnart” (dark green/pink) vs. “SWK001” (light green/red) analysed in ESI(–) mode. (**A**) PCA scores plots of all the samples showing the infected and control groups as indicated. (**C**) PCA scores plot illustrating all infected samples from 1–4 d.p.i. for “Dunnart” (**left**) and “SWK001” (**right**). (**B**,**D**) HCA dendrograms (corresponding to PCA plots (**A**) and (**C**), respectively) showing the hierarchical structure of the data, indicating that the control and infected groups for “Dunnart” and “SWK001”, respectively, cluster together and are grouped separately from one another. In (**D**) the infected groups for “Dunnart” are clustered separately to the left and the infected groups of “SWK001” to the right. The unsupervised modelling tools allowed a comprehensive overview of the data (PCA) and grouped the samples with regard to their treatment-related differences from 1–4 d.p.i. and their natural clustering based on cultivar-dependent variation (HCA).

Principal components analysis (PCA) is a multivariate technique that is used to explore complex datasets by reducing the multi-dimensionality of the data and thereby revealing structures, trends or groupings that allows for biological interpretation of the data [35]. Here, the principal components illustrated treatment- and cultivar-related groupings in the PCA models that revealed the underlying structures and properties of the data (Figure 3A,C). The sample groupings indicate that the “Dunnart” and “SWK001” cultivars had different metabolic responses when treated with *Ps-c*. The “Dunnart” cultivar clustered separately from “SWK001” in both the infected and non-treated control groups, again reiterating treatment and cultivar-related differences (Figure 3A). The negative controls (refer to Section 4.3) clustered with the vehicle controls (Supplementary Figure S4), meaning that there were no significant differences between the two control groups, i.e., the underlying metabolic profiles are similar compared to the infected groups. Therefore, only the non-infected vehicle control groups are further presented throughout the study and referred to only as control. Figure 3B illustrates the PCA model showing differential clustering between the two treated groups (“Dunnart” and “SWK001”) over time (1–4 d.p.i.).

Hierarchical cluster analysis (HCA) graphically presents clusters of the high-dimensional data in the form of a dendrogram based on dissimilarity and similarity between the samples [36]. In this study it was used to evaluate whether groupings emerge from the data based on treatment and/or cultivar-related differences. The bottom-up model (Figure 3B,D) employs an algorithm to cluster each observation based on their similarities/differences and then iteratively combines the most comparable clusters at each step [37]. The computed HCA models show distinct groupings corresponding to the control and infected samples among the “Dunnart” and “SWK001” cultivars (Figure 3B). Treatment-related (control vs. treated) and time-related groupings were additionally formed within each major cluster of the two respective cultivars (Figure 3D). To better interpret the biochemical differences revealed by PCA and HCA in oat responding to *Ps-c* infection, a supervised modelling method, orthogonal partial least squares discriminant analysis (OPLS-DA) was used.

**Figure 4:**
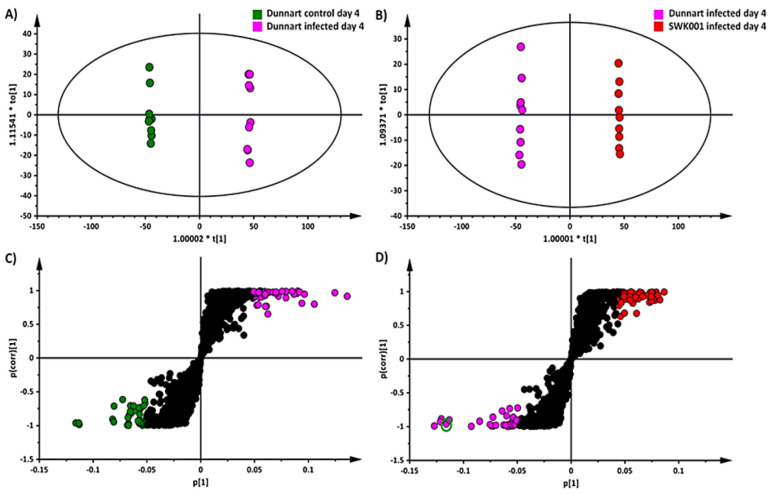
An OPLS-DA model of the two infected cultivars: “Dunnart” and “SWK001”. (**A**) An OPLS-DA scores plot summarising the relationship among different datasets to visualise group clustering between the control and infected “Dunnart” groups at 4 d.p.i. based on their leaf-extracted metabolic profiles obtained in ESI(–) MS mode (R^2^ = 0.999, Q^2^ = 0.995, CV-ANOVA *p*-value = 1.89349 × 10^−15^). (**B**) OPLS-DA scores plot illustrating the relationship among the infected “SWK001” and “Dunnart” plants at 4 d.p.i. based on their leaf-extracted metabolic profiles obtained in ESI(−) MS mode (R^2^ = 0.999, Q^2^ = 0.996, CV-ANOVA *p*-value = 1.06365 × 10^−14^). (**C**) The corresponding OPLS-DA loadings S-plot of (**A**). The pink and green circles indicate the values situated far out [1] (*p* > 0.05, <−0.05 and *p*(corr) >0.5, <−0.5) in the S-plot, representing statistically significant ions that are possible discriminatory variables between the control and infected “Dunnart” groups. (**D**) The corresponding OPLS-DA loadings S-plot of (**B**). The pink and red circles indicate the values situated far out [1] (*p* > 0.05, <−0.05 and *p*(corr) >0.5, <−0.5) in the S-plot, indicating statistically significant ions that are possible discriminatory variables between the infected “SWK001” and “Dunnart” plants. The green circle indicates the selected variable (avenanthramide A) for which validation models are shown in Figure 5.

**Figure 5:**
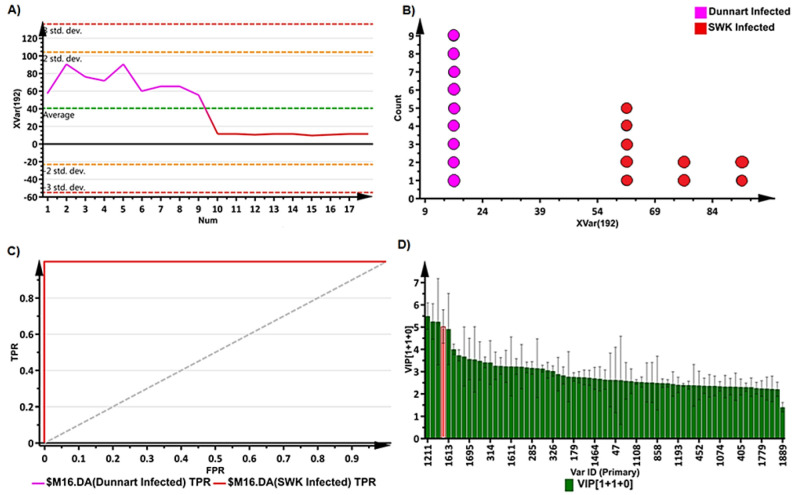
S-plot and OPLS-DA representative validation models. (**A**) Variable trend plot illustrating the selected variable, avenanthramide A, that displayed changes among the “Dunnart” and “SWK001” infected groups at 4.d.p.i. This metabolite is illustrated in red on the VIP plot (**D**) and circled on the S-plot (Figure 4D). (**B**) Dot plot illustrating strong discrimination between infected groups of the “SWK001” and “Dunnart” cultivars for the selected variable (avenanthramide A) as there is no overlap between the groups. (**C**) Receiver operator characteristic (ROC) plot separating the infected groups at 4 d.p.i. for the OPLS-DA model (Figure 4B). The ROC graph is a representation of the performance of the binary classifier. As the curves passes through the top left corner, a model with perfect discrimination is confirmed as having 100% sensitivity and 100% specificity. (**D**) A variable importance for the projection (VIP) plot of Figure 4B, illustrates each variable, its importance and how it contributes to the discrimination of the two groups.

OPLS-DA was applied as a binary classifier that aids in extracting discriminatory variables underlying differential groups [33,38]. The supervised method ensures separation in the scores-space between different experimental groups, as illustrated (Figure 4A,C). The infected and vehicle control groups were used for sample classification for the “Dunnart” cultivar using OPLS-DA modelling (Figure 4A) and shows clear separation in the score space, between the control and infected groups. Figure 4B also indicated clear group separation between the “Dunnart” and “SWK001” infected groups. To confirm the validity and reliability of the computed model, these supervised models were validated using a variety of validation methods [39]. Cross-validation analysis of variance (CV-ANOVA) was used to test the models’ reliability, with significant models having *p*-values of <0.05. Furthermore, the OPLS-DA models’ performance was assessed using receiver operating characteristic curve (ROC) models, with perfect classification represented by the ROC curve passing through the top left corner, indicating perfect sensitivity and specificity (Figure 5C). Finally, permutation tests revealed that the OPLS-DA models were statistically superior to the generated permutation models, with the original OPLS-DA model having a higher R^2^ and Q^2^ value (Supplementary Figure S5).

The OPLS-DA loadings S-plots (Figure 4C,D) were used to evaluate and select statistically discriminatory variables (ions) among the treatment and control groups of the two cultivars, as well as between the treated (infected) groups of both cultivars. OPLS-DA models along with their corresponding loadings S-plots were constructed for the control vs. treated groups, time points and infected “SWK001” vs. “Dunnart” (18 in total for each cultivar—not shown). Variable trends (Figure 5A), dot plots (Figure 5B) and variable importance in projection (VIP) plots (Figure 5D), were used to assess the statistical significance and discriminability of the potential markers acquired from the S-plots. The variable trend plot illustrates how the potential markers discriminate between two groups. A dot plot (Figure 5B) was used to evaluate each selected variable from the respective S-plots. It computes each observation as a unit and subsequently sorts each into “bins” that indicate sub-ranges. There was no overlap between the strong discriminating variables [40,41] as illustrated in Figure 5B. The VIP plots illustrate scores in the form of a column chart, thereby providing a means to assess the importance of the variables in explaining how the X and Y variables correlate to one another [42]. S-plot variables with VIP scores >1.0 and no overlap between groups (illustrated by the trends and dot plots) were selected for further investigation (Table 1).

### 2.4. Metabolic Profiling of *Ps-c* Induced Changes in Infected Oat Leaves 

For biochemical interpretation of metabolic changes in the leaf tissue of the tolerant vs. susceptible oat cultivars responding to infection by *Ps-c*, as graphically illustrated by the chemometric models, the statistically selected discriminatory metabolites from the loadings S-plots were annotated and putatively identified (Table 1) and interpreted for their potential biological roles in oat defence against *Ps-c*. The metabolites were annotated as described in Section 4.8 and classified into the following metabolite groups: amino acids, phenolics, phenolic amides, fatty acids, flavonoids, alkaloids, terpenoids, lipids, saponins and plant hormones. Induced changes across these metabolite classes in the two cultivars were metabolically characterised and showed alterations involved in the plant response to the bacterial pathogen. 

**Figure 6:**
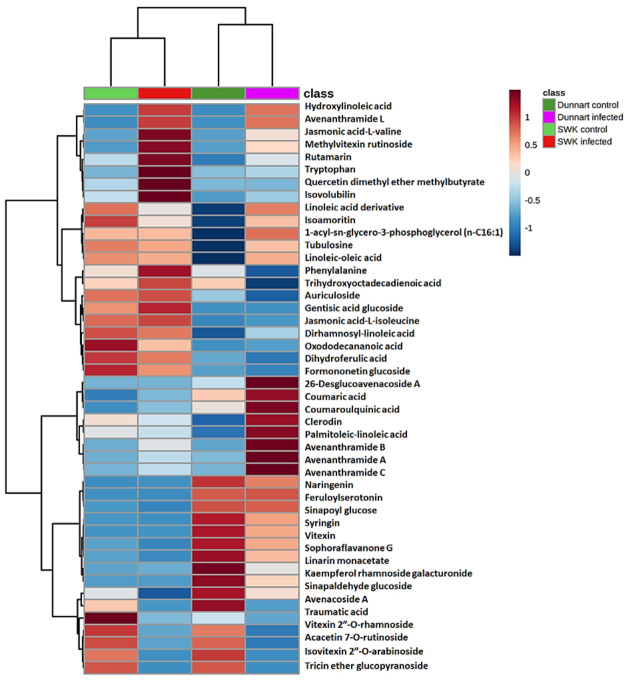
Heatmap analysis of individual peak intensities of the putatively identified discriminatory metabolites from oat leaves treated with *Ps-c.* The map was constructed (using the Pearson distance and Ward’s linkage rule) to illustrate infected and control groups of the two respective cultivars, “Dunnart” and “SWK001”. After *Pareto*-scaling the data, the mean peak intensities of each annotated metabolite are displayed. Higher than average values are shown in brown, while lower values are shown in blue, with each row representing discriminant features and each column representing cultivars and treatment groups, respectively.

With heatmap analysis (Figure 6), the amount and presence of the respective metabolites in various treatment and control groups for the respective cultivars were visualised using data visualisation tools. Heatmaps were created using statistical analysis tools available on MetaboAnalyst [43] by utilising the average integrated peak areas of the individual metabolites. The infographic shows clear differences among the treated and non-treated groups for the respective cultivars. For example, the discriminant features/chemical profiles of the two cultivars becomes obvious and readily explains the observed tolerant (“Dunnart”) vs. susceptible (“SWK0001”) phenotypes. These profiles could demonstrate to be useful in providing information on the chemical basis of disease resistance in oat plants against *Ps-c*, and identify metabolic markers associated with resistance or susceptibility traits. 

**Figure 7:**
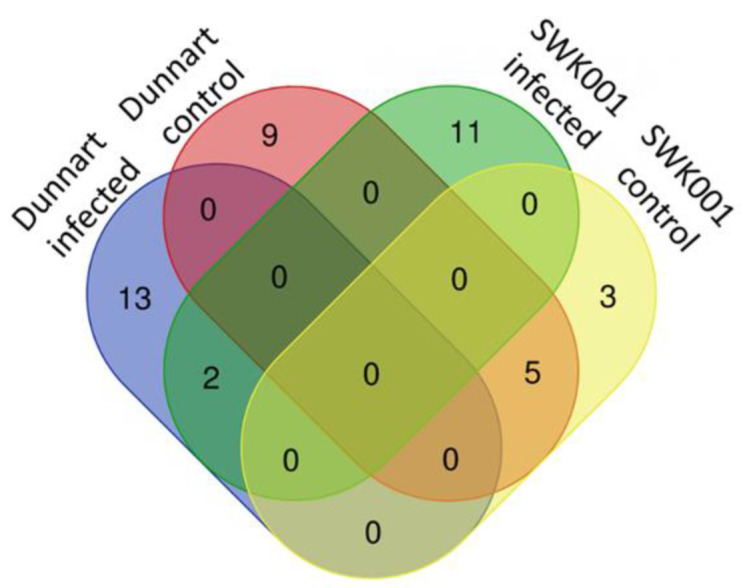
Venn diagram displaying the partial overlap and differences of the statistically significant discriminatory metabolites selected from the OPLS-DA models. Extracts from the infected and control groups of the respective cultivars (tolerant “Dunnart” and susceptible “SWK001”) are compared. The numerical values in the diagram represents the discriminatory metabolites (Table 1) that are unique to the respective cultivars or treatments and conversely, also shared between the cultivars or treatments.

Amongst the identified metabolites (Table 1), the differential metabolic profiles between the treated and control groups based on the discriminatory ions present in the hydromethanolic extracts were as follows: infected “Dunnart” had 2 flavonoids, 2 phenols, 2 fatty acids, 4 phenolic amides, 2 alkaloids, 1 lipid and 1 saponin (26-desglucoavenacoside A) compared to the “Dunnart” control that showed a metabolic profile containing 9 flavonoids, 2 phenols, 1 fatty acid and 1 saponin (avenacoside A) as potential signatory biomarkers. Infected “SWK001” presented a metabolic profile containing 3 flavonoids, 2 phenols, 4 amino acids and 3 fatty acids as discriminatory ions. In comparison, the “SWK001” control showed a metabolic profile generating 5 flavonoids, 1 phenol, 1 fatty acid and 1 saponin (avenacoside A) as potential signatory biomarkers. The Venn diagram (Figure 7) was constructed based on the differential metabolic profiles and illustrates partial overlap and clear distinctions across the cultivars and the treated and control groups.

When compared to the controls, the tolerant “Dunnart” cultivar had a number of metabolites that were particularly discriminatory for the infected group (coumaric acid, traumatic acid, avenanthramide A, B, C and L, coumaroylquinic acid, feruloylserotonin, clerodin, tubulosine, 1-acyl-sn-glycero-3-phosphoglycerol (n-C16:1), isoamoritin, dirhamnosyl-linoleic acid, palmitoleic-linoleic glucoside and 26-desglucoavenacoside A) as illustrated in the heatmap (Figure 6). These metabolites are thus potential markers for the defence response of this particular cultivar to *Ps-c.* By comparison, the infected susceptible “SWK001” cultivar groups showed several discriminatory metabolites compared to the controls (phenylalanine, tryptophan, traumatic acid, hydroxylinolenic acid, jasmonic acid-valine, gentisic acid glucoside, jasmonoyl-isoleucine, avenanthramide L, trihydroxyoctadecadienoic acid, rutamarin, quercetin dimethyl ether methylbutyrate, formononetin glucoside malonate and isovolubilin), making these metabolites possible metabolic markers for the response of this cultivar to *Ps-c*. 

**Figure 8:**
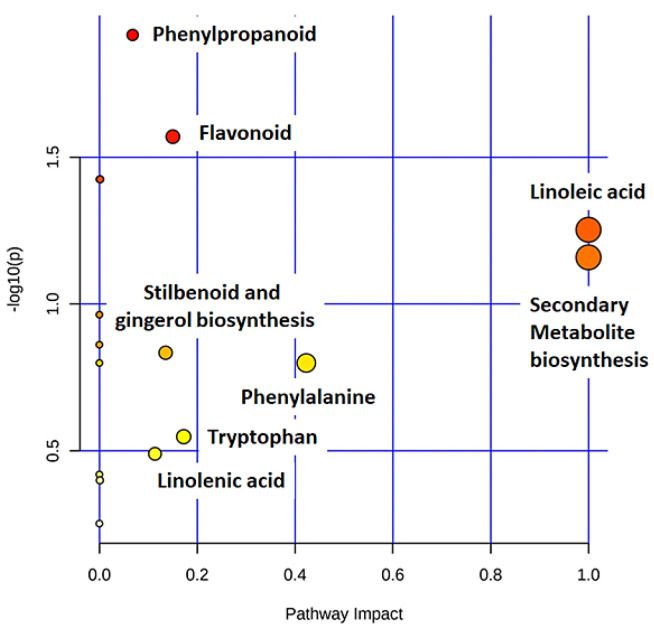
Pathway analysis summary of all MetaboAnalyst-computed metabolic pathways displayed according to their significance or pathway impact. The graphic illustration shows all the matched pathways arranged by *p*-values (*y*-axis; pathway enrichment analysis) and the pathway impact values (*x*-axis; pathway topology analysis). Each node’s colour corresponds to its associated *p*-value, and node sizes are determined by their impact values. Pathways with high impact: linoleic acid (C18:2, n-6) pathway and the general secondary metabolite biosynthesis pathway are illustrated, and furthermore the pathways with high statistical significance were: phenylpropanoid and flavonoid biosynthesis pathways.

**Figure 9:**
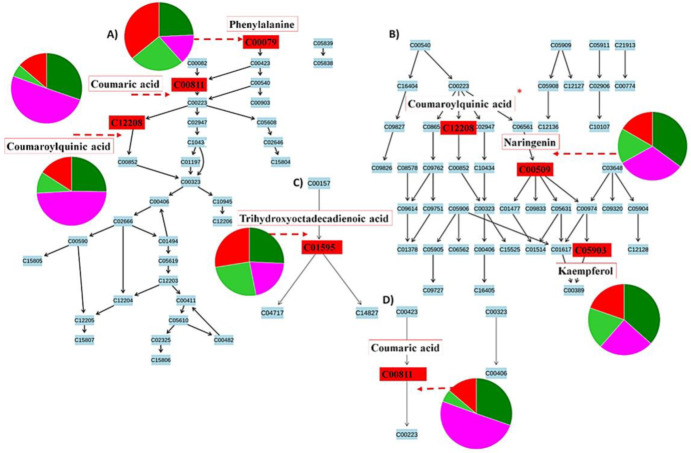
Pathways flagged from metabolomics analysis using MetaboAnalyst software. Signatory metabolites involved in each pathway are illustrated in the form of pie charts according to their relative intensities and presence across the different cultivars and treated groups. (**A**) The phenylpropanoid pathway, (**B**) the flavonoid pathway overlapping with the phenylpropanoid pathway (*), and (**C**) the linoleic acid pathway that showed the highest impact after pathway enrichment analysis, along with (**D**), the general secondary metabolite biosynthesis pathway. Some limitations in MetaboAnalyst prevented the mapping of all annotated metabolites (Table 1). The different colours indicate “Dunnart” infected (pink), “Dunnart control” (dark green), “SWK001” infected (red) and “SWK001” control (light green). The metabolites synthesised via the phenylpropanoid-and flavonoid pathways are some of the most widely occurring secondary metabolites that are involved in plant development and defence against abiotic and biotic stresses, such as phenolics, flavonoids, coumarins and lignin [45,46]. Both pathways are initiated with the conversion of phenylalanine to *p*-coumaroyl-CoA and have some overlap, as shown (*) in Figure 9B. The presence and distribution of the phenolic compounds, from both the phenylpropanoid and flavonoid pathways, across the plant kingdom at a cellular, tissue and organ level emphasises the vast biological and biochemical functions important to the survival of plants [47,48]. Phenolics have been known to play important roles in plant-pathogen interactions as either pre-formed (phytoanticipins) or induced anti-pathogenic molecules (phytoalexins) [49]. Linoleic acids (C18:2) are unsaturated fatty acids that are prevalent in plant membranes, hence making them important for plant structure and maintaining water permeability and, additionally, are involved in the formation of jasmonate, which act as signalling molecules in response to tissue damage caused by insects, pathogens, herbivores or mechanical stress [50].

**Figure 10:**
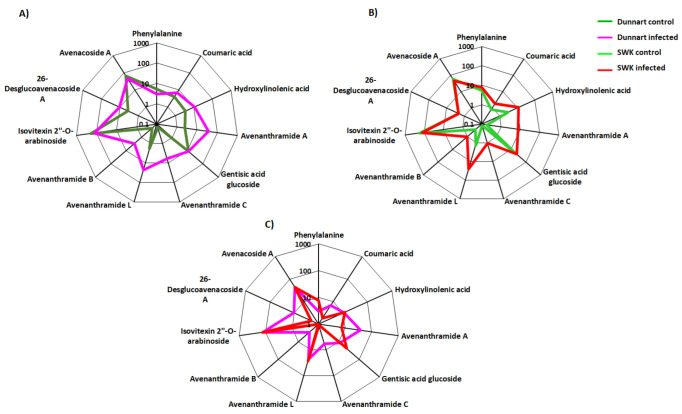
Radar charts illustrating relative intensities of selected biomarker metabolites across the different treated and control groups of the oat cultivars. (**A**) “Dunnart” infected vs. control, (**B**) “SWK001” infected vs. control and (**C**) “Dunnart” infected vs. “SWK001” infected. The relative peak intensities were averaged and illustrated as log-transformed values. The Venn diagram displays the partial overlap and differences of the statistically significant variables from the treated and control groups of the two cultivars. The numerical values illustrate the metabolites that were unique to the respective cultivars and treatments (Figure 7). The “Dunnart” infected group shows the presence of several metabolite classes (phenols, fatty acids, flavonoids, phenolic amides, alkaloids, lipids and saponins) with the three unique phenolic amides (avenanthramide A-C) and 26-desglucoavenacoside A being among the metabolites involved in plant defence for this cultivar. Conversely, the “SWK001” cultivar lacked these phenolic amides and the 26-desglucoavencoside A saponin as discriminant ions. The only overlap illustrated between the defence responses of “SWK001” and “Dunnart” are the presence of traumatic acid and avenanthramide L as discriminatory variables for both these cultivars.

Coumaric acid, as an example, is shown as a discriminatory metabolite for the “Dunnart” infected group. It serves as an entry point into the phenylpropanoid metabolic pathway of secondary metabolites (Figure 8) which is additionally confirmed by Figure 9A,D (pie charts), displaying the pathways in which this metabolite is involved, as well as how it is distributed between cultivars and treatments as shown in Figure 10 (radar charts). When comparing the metabolites involved in the response to the bacteria, the increasing presence and abundance of avenanthramides from 1–4 d.p.i. among the two cultivars were illustrated using colour coded PCA scores plots, where “Dunnart” contained avenanthramide A–C and L at an increasing presence from 1–4 d.p.i. in comparison to “SWK001” that contained only avenanthramide L (Supplementary Figure S6). Based on the distribution and presence of these metabolites, it is clear that the adaptive immune responses of the two cultivars towards *Ps-c* were differentially reflected at the metabolome level. Furthermore, the metabolomics analyses allowed a rapid and sensitive means of detecting the presence of specific secondary metabolites among the different treated cultivars, making it useful for biomarker discovery related to plant-pathogen interactions.

Defence-related metabolites do not work in isolation but are rather interconnected to each other in different metabolic pathways. Metabolic pathway mapping was used to uncover the most relevant pathways involved in oat responses to *Ps-c* infection for biochemical interpretation of the post-treatment metabolic perturbations in oat plants. To further analyse the metabolomic reprogramming induced by *Ps-c* infection, metabolomics pathway analysis (MetPA) was performed (MetaboAnalyst 4.0). This highly sensitive web-based tool is useful in the analysis and visualisation of metabolomic data and can detect subtle changes among different metabolites. As a result, biological pathways can be generated based on these concentration changes, or alternatively from a compound list with known KEGG (Kyoto Encylopedia for Genes and Genomes) or HMDB (Human Metabolome Data Base) compound identifiers (IDs) [43,44]. The computed metabolic pathways are presented conferring to pathway significance or impact as shown in Figure 8. The most significant pathways (displayed on the *y*-axis) were the phenylpropanoid-and flavonoid pathways, whereas the most impactful pathways (displayed on the *x*-axis) were the linoleic acid-and (in general) the secondary metabolite biosynthesis pathway.

In addition to the heatmaps (Figure 6), differences regarding the relative intensities of identified discriminatory metabolites were also explored among the treated and control groups using colour coded PCA scores plots (Supplementary Figure S6), pie charts (Figure 9) and radar charts (Figure 10). Pie charts (Figure 9) depict the relative intensities of the respective metabolites among the various pathways. The general secondary metabolite biosynthesis pathway (Figure 9D) shows *p*-coumaric acid converted to *p*-coumaroyl-CoA and caffeoyl-CoA converted to feruloyl-CoA, the two precursor metabolites involved in the synthesis of avenanthramides. Avenanthramide biosynthesis is initialised by the enzymatic synthesis of *p*-coumaric acid from phenylalanine by phenylalanine ammonia lyase (PAL) and cinnamate 4 hydroxylase (C4′H) or directly from tyrosine by tyrosine ammonia lyase (TAL). *p*-Coumaric acid is then transformed into its activated CoA thioester by 4CL, to condense with 5-hydroxyanthranilic acid, catalysed by hydroxyanthranilate *N*-hydroxycinnamoyl transferase (HHT), to form avenanthramide A. Conversely, the *p*-coumaroyl-CoA is often first converted to *p*-coumaroyl shikimate or quinate, in which case it becomes hydroxylated by *p*-coumaroyl CoA ester 3′-hydroxylase to form caffeoyl-CoA. Subsequently, the caffeoyl-CoA is condensed with 5-hydroxyanthranilic acid in the presence of HHT to form avenanthramide C. Finally, avenanthramide C is methylated by the caffeoyl-CoA O-methyltransferase (OMT) enzyme to form avenanthramide B [51,52,53]. 

VIP scores plots (Supplementary Figure S7), generated using MetaboAnalyst, indicates the key discriminatory metabolites with a VIP score of >0.5 which are considered significant in discriminating between the *Ps-c* treatment and controls among the two cultivars. Some examples can be seen, as in the case with avenanthramide L (Figure S7), which presented as a discriminatory feature in the “Dunnart” and “SWK001” treated groups, with “SWK001” showing a greater abundance. As before, the averaged peak intensities of each metabolite were combined to create radar charts/radial plots (Figure 10), this time comparing metabolic alterations among the individual cultivars in response to bacterial infection of the leaf tissue. A variety of metabolites are presented in the corresponding radar plots based on their log-transformed averaged peak intensities. In Figure 10A,B clear differences and correlations can be seen between “Dunnart” and “SWK001” treated and control groups as well as between “Dunnart” and “SWK001” treated groups (Figure 10C). Avenanthramides A, B and C, for example, are shown to be least abundant in “SWK001” and most abundant in “Dunnart” (Figure 10C). These charts are therefore informative in differentiating among the various cultivars and treatments based on the respective discriminatory metabolites. 

## 3. Discussion

Immune surveillance by the host involves both extracellular-and intracellular recognition (PAMP-triggered immunity or PTI, and effector-triggered immunity (ETI). In turn, some pathogens are able to counter activated plant defences through the injection of suppressors, resulting in Effector-triggered susceptibility (ETS) [23]. In general, the sum of PTI and ETI, minus inhibitory effects due to ETS (taking timing and extent of responses into account), would determine the phenotypic outcome as resistant, tolerant or susceptible. 

Metabolomics investigations into plant-microbe interactions have opened avenues to examine the intricate details of activation and re-direction of plant metabolism as an adaptive strategy upon initiation of defence in *Sorghum bicolor* against infection by *Burkholderia andropogonis* [54] and *Colletotrichum sublineolum* [55]. In previous studies on tomato, *Solanum* *lycopersicum* [56], metabolomics has been applied for comparative metabolic phenotyping to identify metabolic signatures linked to varied response capacities predicted by phenotypic plasticity in cultivars with different resistance capabilities [57]. Similar to this study, the phenylpropanoid-and associated pathways were revealed as the fundamental hub of induced defences against *Ralstonia solanacearum* [56] and *Phytophthora capsici* [58]. Increased quantities and diversity of metabolites linked to defence suggested cultivar-specific differences in the mode and speed of resource redistribution. 

Changes in the composition of the oat leaf metabolome were found to indicate an inducible phenotype in the host plant when inoculated with *Ps-c.* A comparison of the differential metabolites that could be positively annotated in leaf extracts from inoculated plants is presented, revealing discriminating metabolic signatures linked to tolerant (“Dunnart”) vs. susceptible (“SWK001”) metabolic phenotypes that underpin defence metabolism and define the defensive capabilities of tolerant vs. susceptible oat cultivars.

Given the dynamic nature of the plant metabolism, qualitative and quantitative differences in specific metabolites or classes within broader metabolomic profiles may have an impact on the outcome of an infection response [58]. These metabolites (Table 1 and explained below) were observed to accumulate in varied amounts and with different accumulation patterns in the leaves of the two cultivars. These patterns suggest that differential reprogramming has occurred over time. This can take the form of high or low accumulation at specified time points, indicating early-, late or oscillatory responses. Infected plants re-adjust their metabolomes toward inducible defence responses to limit pathogen entry and multiplication, according to the time-dependent reprogramming [58]. 

Among the triggered alterations, plant hormones were identified namely jasmonates (jasmonoyl-isoleucine, jasmonic acid-valine) and traumatic acid. Jasmonates are produced as a signalling hormone in response to pathogen attack and causes the plant to reconfigure its metabolism to produce potent defensive secondary metabolites [59]. This process is facilitated by the jasmonate signalling pathway that leads to the rapid accumulation of jasmonic acid (JA) and its bioactive form jasmonoyl-isoleucine, which presented as a discriminatory metabolite for treated “SWK001”, contrary to the treated “Dunnart” where it did not present as discriminatory. This can possibly be ascribed to differences in the defence responses active in the cultivars. The timing and extent of jasmonate responses depend on the plant, tissue type, type of stress and the environmental conditions [60]. “Dunnart” could have therefore, had a lower abundance due to an earlier mobilisation of the pathogen-triggered signals and subsequent conversion and deactivation of the bioactive jasmonoyl-isoleucine into a non-active conjugate when it was no longer needed. These signalling hormones cause transcriptomic and metabolic reconfigurations that aid in plant defence responses [61,62]. Many different jasmonate precursors and derivatives have been known to exhibit biological activity, including jasmonic-amino acid conjugates [63], of which two were identified in this study in response to *Ps-c* infection. Traumatic acids are organic compounds that are often referred to as plant growth and development regulators that participate in the regulation of plant metabolism [64]. Traumatic acid is generally classified as a wound hormone and has been found to accumulate in large quantities around plant wounded areas [65]. It has, however, also been identified as an induced resistance metabolite for barley against Fusarium head blight disease [66] and its detection in oats due to *Ps-c* infection suggest an additional defensive role. Traumatic acid and JA biosynthesis occurs from the octadecanoic pathway via either linoleic acid or linolenic acid [50,65,67]. These are both 18-carbon unsaturated fatty acids precursors that were also identified among the discriminatory metabolites. The synthesis of these hormones initiates a physiological response in plants to increase the production of defence related compounds like phenolics, flavonoids, alkaloids and terpenes [68]. 

Phenolic acids are produced and accumulate in plant tissues in response to stress and/or pathogen attack, where they act as protective agents against invading organisms like insects, fungi, nematodes, and bacterial pathogens [69]. Phenolic acids are produced by the phenylpropanoid pathway from phenylalanine through deamination, hydroxylation, and methylation [70]. A unique group of phenolic acid amides were identified as discriminatory in the treated groups of both “Dunnart” and “SWK001” and were absent in the respective controls. These phytoalexins, known as avenanthramides, are unique to oat plants and consist of an anthranilic acid bound to a hydroxycinnamic acid via an amide bond (Supplementary Figure S8). Over 40 different types of avenanthramides have been identified and classified based on their structure in oat leaves and grains. The most abundant avenanthramides are A, B and C [53,71], which were identified as discriminatory ions/metabolites for the “Dunnart” treated groups. Avenanthramide L was also identified in both the “Dunnart” and “SWK001” treated groups. The biosynthesis of these compounds is initiated by the synthesis of *p*-coumaric acid from phenylalanine, both of which presented as discriminatory metabolites for the treated groups. Hydroxycinnamates (including coumaric-, caffeic-, ferulic-and sinapic acids) are frequently upregulated upon pathogen infection, reiterating their high biological role in plants as antibacterial and antifungal metabolites [69,72]. The footprint of hydroxycinnamic acids is frequently seen in metabolite profiles as the associated conjugates and derivatives [56,72]. Similarly, avenanthramides are produced in response to pathogen infection or when oat leaves are treated with various elicitors [73]. As phytoalexins, these inducible compounds function as both chemical defence and as substrates for the reinforcement of cell walls in physical defence upon exposure to pathogens [74]. The greater abundance and presence of these compounds in “Dunnart”, could have contributed to the defence response and tolerance of the cultivar to the bacteria, in contrast to the “SWK001” cultivar that only presented avenanthramide L as a discriminatory metabolite and showed greater susceptibility in response to the treatment and resulted in severe symptom development. 

As nitrogen containing metabolites, alkaloids form part of important secondary metabolites that play a crucial role in plant defence, especially as antimicrobial compounds [9]. Alkaloids are synthesised from amino acid precursors such as aspartate, lysine, tyrosine, and tryptophan. These bioactive compounds have exceptional biological activities mostly attributed to the ability to form hydrogen bonds with proteins, enzymes and receptors, due to the presence of a nitrogen atom (proton accepting) and amine hydrogen group(s) (proton donating) [75,76]. Two alkaloids were identified as discriminatory in the “Dunnart” treated groups that possibly contributed to the antimicrobial activity against *Ps-c*. The first is tubulosine, an isoquinoline alkaloid derived from tyrosine and the second feruloylserotonin (also a hydroxycinnamic acid amide) [77,78,79].

Another class of secondary metabolites that greatly contribute to plant defence is the terpenoids. Terpenoids are a large group of phytochemicals that exhibit antimicrobial activity. The majority of terpenoids are antimicrobial due to their ability to inhibit two crucial processes necessary for microbial survival, which includes oxidative phosphorylation and oxygen uptake [80,81]. Here, clerodin, a diterpenoid saponin, was found as a discriminatory metabolite in leaf extracts from the infected “Dunnart” cultivar, but not in the case of “SWK001”. Both cultivars had two saponin compounds that were found to be discriminatory between the treated and control groups (avenacoside A and 26-desglucoavenacoside A). Avenacosides are phytoanticipins that are biologically inactive, and in response to tissue damage or pathogen attack, are transformed into biologically active 26-desglucoavenacosides by an enzyme called avenacosidase [82]. Here, avenacoside A can be seen as showing a decrease in level from the control to the infected groups, with 26-desglucoavenacoside A increasing in the treated “Dunnart” cultivar. In the context of plant defence, it is of interest that desglucoavenacoside A was not detected among the discriminatory metabolites in “SWK001”. Thus, a clear response can be seen as the biologically inactive phytoanticipin was converted to its biologically active form upon treatment with *Ps-c*. The ability of the compound to bind with sterols in the pathogen membrane and disrupt its integrity is the primary mechanism of action against the pathogen. This mechanism is considered to culminate in the creation of transmembrane pores as a result of saponin aggregation with sterol groups, resulting in cell content leakage and, eventually, cell death [82,83,84,85]. 

To summarise, distinct metabolic differences can be seen in the two investigated oat cultivars in response to *Ps-c* infection. Based on the phenotypic and metabolic profiles, the “Dunnart” cultivar showed a greater tolerance to *Ps-c*, which can be attributed to the defence metabolites synthesised by this cultivar in an attempt to limit pathogen spread and symptom development. “SWK001”, on the other hand, showed severe symptom development that resulted in chlorotic wilted leaves, that could be attributed to the lack of defence metabolites that are both adequate (present in high concentrations) and effective (exhibiting anti-microbial activity), ultimately allowing the pathogen to overcome the triggered plant defences and fully infect the leaves.

## 4. Materials and Methods

### 4.1. Oat Plant Cultivation

Seeds of the oat cultivars “Dunnart” (Agricol, Pretoria, South Africa) and “SWK001” (ARC Small Grain Institute, Bethlehem, South Africa) were selected for infection through an initial screening trial (Section 4.2). Seedlings were grown in 10 cm pots (15 seeds per pot) containing germination mixture (Culterra, Muldersdrift, South Africa), and watered twice a week. Greenhouse conditions were used to grow the cultivars which included: a light/dark cycle of 12 h/12 h, with a light intensity of about 84 µmol/m^2^/s and temperature between 25–28 °C. The study was planned to monitor the response of these cultivars to bacterial infection over time, 1–4 days post-inoculation (d.p.i.). The seedlings were grown in triplicate as biological replicates (one pot = one biological replicate) for every time point and the corresponding control groups under the same environmental conditions. Once the plants reached 3-week maturity (seedling stage or three-leaf stage), they were infected (Section 4.3). Following optimisation, the entire experiment was repeated twice. 

### 4.2. Preparation of Pseudomonas syringae pv. coronafaciens 

A culture/strain of *Pseudomonas syringae,* pathogenic on oat, was isolated by Dr. W. Kriel (Starke Ayres Seeds, Bredell, South Africa) and its identity as *P. syringae* pv. *coronafaciens* (including 16S rRNA sequencing-Supplementary Figure S9) was confirmed (Prof. T. Coutinho, Centre for Microbial Ecology, University of Pretoria, South Africa) [15,19]. This *Ps-c* isolate was then grown and maintained on nutrient agar. A colony was picked under sterile conditions in a laminar flow cabinet and grown overnight at 28 °C in nutrient broth on an orbital shaking incubator. The OD_600_ of the overnight culture was measured and diluted with 0.1% Tween 20 and phosphate buffered saline (PBS) to an OD_600_~0.3. The evaluation of the virulence of the *Ps-c* strain on the oat cultivars was determined to be optimal at an OD_600_ of 0.3 based on initial drop-inoculation tests [86] comparing OD_600_ = 0.1, 0.2 and 0.3. The tests were done on inoculated leaf segments kept in a container with high humidity for 5 d. The selection of “Dunnart” and “SWK0001” for further metabolomic investigation as cultivars exhibiting a resistance response vs. a susceptible response, was based on initial visual observation that showed “Dunnart” being able to tolerate the infection over the course of 5 d, and “SWK0001” showing symptoms and susceptible characteristics as soon as 2 d after drop-inoculation, respectively (results not shown). 

### 4.3. Inoculation of Oat Seedlings

At the three-leaf growth stage, oat leaves were inoculated by spraying with the *Ps-c* bacterial suspension (0.1% Tween 20 and phosphate-buffered saline, PBS), diluted to OD_600_~0.3. The non-treated (vehicle) control plants were sprayed with a solution free of the bacteria and the negative control groups were untreated (i.e., not sprayed with either solution) and grown under normal growth conditions. The 50 mL of either the inoculum (containing the bacteria) or the control (0.1% Tween 20 and PBS) solution was evenly sprayed onto the leaves of the treated and non-treated control groups respectively. The plants were then incubated in darkness in an incubator for 1 h to provide 100% relative humidity. Following the 1 h incubation, the plants were removed and another 50 mL of either inoculum or control solution was applied to the treated and non-treated control groups, respectively, and further incubated for 6 h. After incubation the plants were again placed in the same initial conditions as described (Section 4.1). Post-treatment harvesting of plants was done for treated, non-treated and negative control groups at 1, 2, 3 and 4 d.p.i. by harvesting the leaves and immediately snap freezing with liquid nitrogen to quench metabolic activity associated with possible wounding and handling of the tissue. Leaves were kept at −80 °C until metabolite extraction.

### 4.4. Metabolite Extraction and Sample Preparation

The leaf material was quenched in liquid nitrogen before being crushed into a powder with a mortar and pestle. One gram of each sample was weighed out into 50 mL Falcon tubes, followed by the addition of 10 mL 80% cold aqueous methanol (4 °C) (*m/v* ratio of 1:10). The methanol utilised in this experiment was of analytical grade (Rochelle Chemicals, Johannesburg, South Africa). The mixture was then homogenised for 10 s per sample using a probe sonicator (Bandelin Sonopuls, Berlin, Germany) set to 55% power. To avoid cross-contamination, the equipment was cleaned between samples. In a benchtop centrifuge, the homogenates were centrifuged at 5100× *g* for 20 min at 4 °C, next the supernatants were retained and reduced by evaporating the methanol under vacuum to roughly 1 mL by making use of a rotary evaporator set to 55 °C. The concentrated extracts were pipetted into Eppendorf microcentrifuge tubes with a capacity of 2 mL and dried under vacuum in a centrifugal evaporator. After that, 500 μL of 50% aqueous methanol (LC-grade, Romil Pure Chemistry, Cambridge, UK) was added to the dried extracts to reconstitute and dissolve the pellet. The extracts were then filtered (0.22 μm) using nylon syringe filters and injected into 500 μL inserts fitted in chromatography vials, capped and stored at 4 °C until analysis.

### 4.5. Sample Analyses Using Ultra-High-Performance Liquid Chromatography (UHPLC)

An Acquity UHPLC system (Waters Corporation, Manchester, UK) was used to analyse 2 µL of each sample, which separated into its constituents using a binary solvent on an HSS T3 reverse-phase column (Waters Corporation, Billerica, MA, USA; 2.1 × 150 mm × 1.7 µm), thermostatted at 60 °C. MilliQ water and acetonitrile (Romil Chemistry, Cambridge, UK), were used as solvents, with both containing 0.1% formic acid (Sigma, Munich, Germany) and 2.5% isopropanol (IPA, Romil, Cambridge, UK). The run was set to 30 min per 2 μL injection with an elution gradient performed using a binary solvent system consisting of 0.1% aqueous formic acid (solvent A) and 0.1% formic acid in acetonitrile (Romil Pure Chemistry, Cambridge, UK; solvent B) at a flow rate of 0.4 mL/min. Initially the conditions were set to 95% A and 5% B which was held for 1 min. At 25 min, a gradient was used to change the chromatographic conditions to 10% A and 90% B, which was then modified to 5% A and 95% B at 25.10 min. Conditions were maintained for 2 min before being switched back to the starting conditions at 28 min. Before the next injection, the analytical column was allowed to equilibrate for 2 min. To monitor the state of the LC–MS equipment and assess the the reliability and reproducibility of each analysis, pooled quality control (QC) samples were included in each [87]. In addition, blank samples (50% MeOH) were added in the run at random to measure the background noise. To account for analytical variability and have the minimal number of required replicates for metabolomic investigations that involve multivariate data analyses, each sample was analysed in triplicate (analytical/technical replicates), which, when combined with three biological replicates, yielded *n* = 9.

### 4.6. Quadrupole Time-of-Flight Mass Spectrometry (q–TOF–MS) 

To detect and capture metabolites data in both positive and negative electrospray ionisation (ESI) modes, a high definition SYNAPT G1 Q-TOF mass spectrometer (Waters Corpora-tion, Manchester, UK) was used in conjunction with the chromatography system. MassLynx XS^TM^ (Waters, Manchester, UK) was used as the controlling software for the system. Leucine encephalin (554.2615 Da) was employed as the “lockmass” calibrant (50 pg/mL, [M + H]^+^ = 556.2771 and [M − H]^−^ = 554.2615), which was continuously sampled every 15 s, yielding an average intensity of 350 counts per scan in centroid mode. The mass accuracy window was 0.5 Da, while the typical mass accuracy ranged from 1 to 3 mDa. The capillary and sample cone voltages were set to 2.5 kV and 30 V, respectively. The desolvation temperature was 450 °C, with a source temperature of 120 °C, a cone gas flow of 50 L/h, and a desolvation gas flow of 550 L/h. A scan time of 0.1 s was used with a *m/z* range of 50–1200. At a flow rate of 700 L/h, high-purity nitrogen was employed for desolvation, collision, and cone gas. Data was collected using five different mass spectrometry elevated (MS^E,^) collision energies (a data-independent acquisition approach), ranging from 0 to 50 eV, to trigger fragmentation of the initial ions and capture as much structural information as possible for later structural interpretation and metabolite identification [57,88].

### 4.7. Data Analysis

The MarkerLynx XS^TM^ version 4.1 (Waters Corporation, Manchester, UK) application manager was used to examine and process the data sets received. The software employed the patented ApexTrack algorithm. The following processing parameters were used: retention time (Rt) ranges from 2–23 min, while the *m/z* ranged from 50–1200 Da. The Rt and mass windows were each set to 0.20 min and 0.05 Da, respectively. The intensity threshold was set to 100 counts and the mass tolerance to 0.05 Da. For multivariate data analysis (MVDA), the rectified data matrices were then exported to “soft independent modelling of class analogy” (SIMCA-version 15) software (Umetrics, Umea, Sweden). To reduce the dimensionality of the data sets and to study the underlying structures and properties of the data, unsupervised models, such as principal component analysis (PCA) and hierarchical clustering analysis (HCA) were utilised. To compare the two cultivars and find discriminating ions, supervised orthogonal projection to latent structures discriminant analysis (OPLS-DA) was utilised. Validation approaches were then applied to validate the OPLS-DA models and included cross-validated analysis of variance (CV-ANOVA) and receiver operator characteristic (ROC) analysis [33,38,39].

### 4.8. Metabolite Annotation and Semi-Quantitative Comparison

The MarkerLynx^TM^ software generated possible elemental compositions and accurate masses that were used along with fragmentation patterns to identify the respective metabolites. Each ostensibly recommended empirical formula was exported and searched in a variety of databases, including MetaCyc [89], plant metabolic network (PMN) [90], ChemSpider, mass bank of North America [91], Dictionary of Natural Products [92] and KEGG (the Kyoto Encyclopaedia of Genes and Genomes) [93]. Unless otherwise stated, metabolites were putatively identified as outlined by the Metabolomics Standards Initiative (MSI) to level 2 [94].

The generated matrixes of the annotated metabolites were exported from MarkerLynx XS™ as .csv files. MetaboAnalyst 4.0 is an online platform that can be used to analyse metabolomics data statistically, functionally and integratively (www.metaboanalyst.ca, accessed on 11 May 2021). In this study it was utilised for the visualisation of the MS files which contained the *m/z*, Rt and peak intensities of metabolites separated by chromatography and detected as ions by mass spectrometry. Data processing, integrity, missing values, filtering, and normalisation were performed on MetaboAnalyst followed by *Pareto*-scaling before statistical analyses to reduce variance within the features. Heatmap analyses employing a Pearson distance measure and the Ward clustering method (www.metaboanalyst.ca) were utilised to compare the magnitude and occurrence of the detected metabolites among the various cultivars and treatments [43,95]. Partial least square-discriminant analysis (PLS-DA) was utilised to unravel the data in MetaboAnalyst as a means of comparing and visualising the relative abundances of the identified metabolites across the various cultivar treatments. The main discriminatory metabolites identified with VIP scores plots, had a VIP score of >0.5 which are considered significant when comparing the *Ps-c* treatment and controls among the two cultivars. Additionally, radar/radial plots were constructed based on the means and relative intensities displayed as log-transformed values to better visualise changes among the discriminating metabolites linked to the defence responses of the two cultivars (Section 2.4).

## 5. Conclusions

A systems biology approach for understanding the biochemical and molecular mechanisms underlying plant immune responses has become vital in the search for innovative techniques to aid in plant defence against continually evolving pathogens. Oat plants have been greatly underrated, with little research regarding the metabolic response of this cereal to pathogenic threats. Therefore, this study reports on the metabolic markers and mechanisms involved in oat response to pathogen attack. These markers indicate gradations in cultivar-related defences and would thus provide insight into the tolerant and/or susceptibility events that are involved under biotic stress. 

Among the findings, some intriguing correlations can be drawn between the metabolic profiles of the cultivars and their natural variance in comparison to their tolerant and susceptible defence responses to *Ps-c* exposure. When comparing the “Dunnart” and “SWK001” cultivars under normal conditions (controls), distinct metabolic profiles with some overlap were observed. Avenacoside A, for example, was shown to be discriminatory for both cultivars under healthy conditions, with the “Dunnart” cultivar having a higher relative abundance as revealed via heatmap analysis. An increasing amount of 26-desglucoavencoside A, which is the biologically active counterpart, was detected after exposure to *Ps-c*, and was identified as a discriminant metabolite for “Dunnart”. In the case of the “SWK001” cultivar, however, no substantial convergence was seen, which could be related to “Dunnart” having a higher abundance of avenacoside A in naïve plants. As a result, prior to stress exposure, the metabolic profiles of the cultivars could potentially aid in predicting the plant’s ability to react and produce a successful defensive capability. 

In this study, a LC–MS untargeted metabolomics method was applied to obtain a detailed understanding of the defensive metabolism of oat plants in response to *Ps-c*. Multivariate data analysis identified signatory metabolites/discriminatory markers from diverse metabolic classes, indicating a broad-based chemical defence response. Moreover, the research showed possible metabolic pathways involved in metabolic alterations in response to *Ps-c*, with phenylpropanoid and flavonoid biosynthesis being most significant, and the most impactful pathways being linoleic-and secondary metabolite (avenanthramide) biosynthesis pathways. The different biological actions of the secondary metabolite classes listed are vital in preventing pathogen infections and preserving the plant under various environmental circumstances. Ultimately, after inoculation with *Ps-c*, an untargeted LC–MS-based metabolomics method can be employed to uncover the underlying metabolic alterations and identify metabolic markers that contribute to the oat defence response. This will aid in obtaining a more comprehensive understanding of the oat metabolome under biotic stress, which can then be used in crop improvement, development and breeding strategies. 

## Figures and Tables

**Table 1:** List of key signatory metabolites extracted and putatively identified from leaves of oat plants treated with *Pseudomonas syringae* pv. *coronafaciens*. These distinguishing metabolites were selected using OPLS-DA S-plots, which were validated with rigorous statistical validation methods (explained in text—Figure 5). The metabolites reported here had VIP scores ˃ 1.0. Increases and decreases are indicated for each treatment where infected (I) and controls (C) were compared for the cultivars “Dunnart”- Dun and “SWK001”-SWK.

Putative Identification	Molecular Formula	*m/z*	Rt (min)	Metabolite Class	Cultivar/Condition			
					Dun (I)	Dun (C)	SWK (I)	SWK (C)
Coumaric acid	C_9_H_8_O_3_	163.038	3.41	Phenolic acid	•	○	-	-
Phenylalanine	C_9_H_10_NO_2_	164.069	1.69	Amino acid	-	-	•	○
Tryptophan	C_11_H_12_N_2_O_2_	203.081	2.66	Amino acid	-	-	•	○
Oxododecanoic acid	C_12_H_22_O_3_	213.147	17.25	Fatty acid	-	-	○	•
Traumatic acid	C_12_H_20_O_4_	227.126	16.93	Fatty acid	•	○	•	○
Naringenin	C_15_H_12_O_5_	271.148	8.37	Flavonoid	○	•	-	-
Hydroxylinolenic acid	C_18_H_30_O_3_	293.211	21.64	Fatty acid	-	-	•	○
Avenanthramide A **	C_16_H_13_NO_5_	298.069	14.04	Phenolic amide	•	○	-	-
Jasmonic acid-valine	C_17_H_27_NO_4_	308.092	18.36	JA conjugate	-	-	•	○
Avenanthramide C **	C_16_H_13_NO_6_	314.065	13.06	Phenolic amide	•	○	-	-
Gentisic acid glucoside	C_13_H_16_O_9_	315.069	1.71	Phenolic acid	-	-	•	○
Jasmonoyl-isoleucine	C_18_H_29_NO_4_	322.202	20.45	JA conjugate	-	-	•	○
Avenanthramide L	C_18_H_15_NO_5_	324.085	15.77	Phenolic amide	•	○	•	○
Trihydroxyoctadecadienoic acid	C_18_H_32_O_5_	327.214	16.68	Fatty acid	○	•	•	○
Avenanthramide B **	C_17_H_15_NO_6_	328.082	14.51	Phenolic amide	•	○	-	-
Coumaroylquinic acid	C_16_H_18_O_8_	337.090	3.42	Chlorogenic acid	•	○	-	-
Feruloylserotonin	C_20_H_20_N_2_O_4_	351.127	6.74	Phenolic amide	•	○	-	-
Rutamarin	C_21_H_24_O_5_	355.159	1.58	Coumarin	-	-	•	○
Sinapaldehyde glucoside	C_17_H_22_O_9_	369.119	13.63	Phenolic	○	•	-	-
Dihydroferulic acid glucuronide	C_16_H_20_O_10_	371.096	5.54	Phenolic	-	-	○	•
Syringin	C_17_H_24_O_9_	371.135	16.02	Phenolic	○	•	-	-
Sinapic acid glucose	C_17_H_22_O_10_	385.116	4.52	Phenolic	○	•	-	-
Auriculoside	C_22_H_26_O_10_	393.175	12.22	Flavonoid	-	-	○	•
Quercetin dimethyl ether methylbutyrate	C_22_H_22_O_8_	413.121	16.7	Flavonoid	-	-	•	○
Sophoraflavanone G	C_25_H_28_O_6_	423.186	11.83	Flavonoid	○	•	-	-
Vitexin	C_21_H_20_O_10_	431.095	10.98	Flavonoid	○	•	-	-
Clerodin	C_24_H_34_O_7_	433.234	22.84	Terpenoid	•	○	-	-
Isovolubilin	C_23_H_24_O_9_	443.133	16.91	Flavonoid	-	-	•	○
Tubulosine	C_29_H_37_N_3_O_3_	474.261	21.13	Alkaloid	•	○	-	-
1-Acyl-sn-glycero-3-phosphoglycerol	C_22_H_42_O_9_P	481.254	22.79	Phospholipid	•	○	-	-
Isoamoritin	C_31_H_38_O_6_	505.255	21.51	Flavonoid	•	○	-	-
Formononetin glucoside malonate	C_25_H_23_O_12_	515.247	14.2	Flavonoid	-	-	•	○
Dirhamnosyl-linoleic acid	C_28_H_48_O_11_	559.311	21.9	Fatty acid	•	○	-	-
Isovitexin 2″-O-arabinoside	C_26_H_28_O_14_	563.139 #	10.33	Flavonoid	○	•	○	•
Vitexin 2″-O-rhamnoside	C_27_H_30_O_14_	577.154	10.75	Flavonoid	○	•	○	•
Acacetin 7-O-rutinoside	C_28_H_32_O_14_	593.149 #	11.39	Flavonoid	○	•	○	•
Kaempferol rhamnoside galacturonide	C_27_H_28_O_16_	607.132	9.32	Flavonoid	○	•	-	-
Linarin monoacetate	C_30_H_34_O_15_	633.181	9.32	Flavonoid	○	•	-	-
Prenylkaempferol diglucoside	C_32_H_38_O_16_	677.207	14.51	Flavonoid	○	•	-	-
Tricin ether glucopyranoside	C_33_H_36_O_16_	689.193 #	13.21	Flavonoid	○	•	○	•
Palmitoleic-linoleic glucoside	C_33_H_36_O_16_	723.382	21.85	Fatty acid conjugate	•	○	-	-
26-Desglucoavenacoside A	C_45_H_72_O_18_	945.481	18.45	Steroidal saponin	•	○	-	-
Avenacoside A **	C_51_H_82_O_23_	1063.539 #	16.58	Steroidal saponin	○	•	○	•

(#) Indicates *m/z* value in positive ESI mode. Closed circles (•) and open circles (○) indicate increases and decreases respectively (positively/negatively correlated to the condition). (-) Indicates metabolites that did not present as discriminatory ions in the respective treatments and cultivars. (**) Metabolite identity was confirmed with an authentic analytical standard (level 1) according to the Metabolomics Standards Initiative (MSI).

## Data Availability

The study design information, LC–MS data, data processing and analyses are reported on and incorporated into the main text. Raw data, analyses and data processing information, and the meta-data are deposited to the EMBL-EBI metabolomics repository—MetaboLights50, with the identifier MTBLS2477 (http://www.ebi.ac.uk/metabolights/MTBLS2477 (accession generated 20 March 2021).

## Supplementary Materials

The following are available online at https://www.mdpi.com/article/10.3390/metabo12030248/s1, Figure S1: Key metabolic components that underlie plant responses to abiotic and biotic stressors. Plant metabolomics is a powerful tool for studying the metabolic mechanisms that trigger and embody plant stress responses to environmental (left) and/or pathogenic (right) threats; Figure S2: Halo blight disease severity index on *Avena sativa* L. inoculated with *Pseudomonas syringae* pv. *coronafaciens*. Example images represent visual observation of disease severity using a 0–8 scale, where 0 = no disease symptoms, 1–3 = slight disease symptoms, 3–6 = moderate disease symptoms and 6–8 = severe symptoms of yellowing and wilting; Figure S3: Typical symptom development of halo blight on “Dunnart” leaves in response to *Ps-c* infection; Figure S4: Principal component analysis (PCA) of the ESI(−) data illustrating the two infected oat cultivars and the respective control groups; Figure S5: An orthogonal projection to latent structures discriminant analysis (OPLS-DA) model of the two infected cultivars, “Dunnart” and “SWK001”; Figure S6: Colour coded PCA score plots showing the presence and increasing abundance of discriminatory ions in the respective treated cultivars, “Dunnart” & “SWK001”, (red- high abundance, blue- low abundance); Figure S7: Variable importance in projection (VIP) scores generated using MetaboAnalyst softwate. Indicated are the top 15 discriminating ions in oat leaves from the respective cultivars “Dunnart” & “SWK001” for their treated and control groups. Metabolites with a VIP score ≥0.5 were considered to be significant in the discrimination between the cultivars; Figure S8: Avenanthramide structures illustrating the core structure and various functional groups unique to each compound; Figure S9: 16S rRNA sequence of *Pseudomonas syringae* pv. *coronafaciens* [19].
