# Supplementary material for: Metabolomic Characterisation of Discriminatory Metabolites Involved in Halo Blight Disease in Oat Cultivars Caused by Pseudomonas syringae pv. coronafaciens
Source: Metabolites. 2022 Mar 16;12(3):248. doi: 10.3390/metabo12030248 (PMC8950619; doi:10.3390/metabo12030248)
Supplement: Supplementary file 1 [file metabolites-12-00248-s001.zip › metabolites-1618031-supplementary.pdf]

metabolites

Metabolomic Characterisation of Discriminatory Metabolites Involved in Halo Blight Disease in Oat Cultivars Caused by *Pseudomonas syringae* pv. *coronafaciens*

Chanel J. Pretorius, Paul A. Steenkamp, Fidele Tugizimana, Lizelle A. Piater and Ian A. Dubery *

Research Centre for Plant Metabolomics, Department of Biochemistry, University of Johannesburg, Auckland Park, Johannesburg 2006, South Africa; 201423600@student.uj.ac.za (C.J.P.); psteenkamp@uj.ac.za (P.A.S.); ftugizimana@uj.ac.za (F.T.); lpiater@uj.ac.za (L.A.P.)

* Correspondence: idubery@uj.ac.za (I.A.D.); Tel.: +27-11-5592401

Supplementary material – All relevant material referred to, but not included in the main text are included in the following supplementary section. All experimental raw data is available upon request from Prof I.A. Dubery at the Department of Biochemistry, University of Johannesburg.

Citation: Pretorius, C.J.; Steenkamp, P.A.; Tugizimana, F.; Piater, L.A.; Dubery, I.A. Metabolomic Characterisation of Discriminatory Metabolites Involved in Halo Blight Disease in Oat Cultivars Caused by *Pseudomonas syringae* pv. *coronafaciens*. *Metabolites* **2022**, *12*, 248. https://doi.org/10.3390/metabo12030248

Academic Editor: Hirokazu Kawagishi

Received: 14 February 2022
Accepted: 10 March 2022
Published: 16 March 2022

Publisher's Note: MDPI stays neutral with regard to jurisdictional claims in published maps and institutional affiliations.

Copyright: © 2022 by the authors. Licensee MDPI, Basel, Switzerland. This article is an open access article distributed under the terms and conditions of the Creative Commons Attribution (CC BY) license (https://creativecommons.org/licenses/by/4.0/).

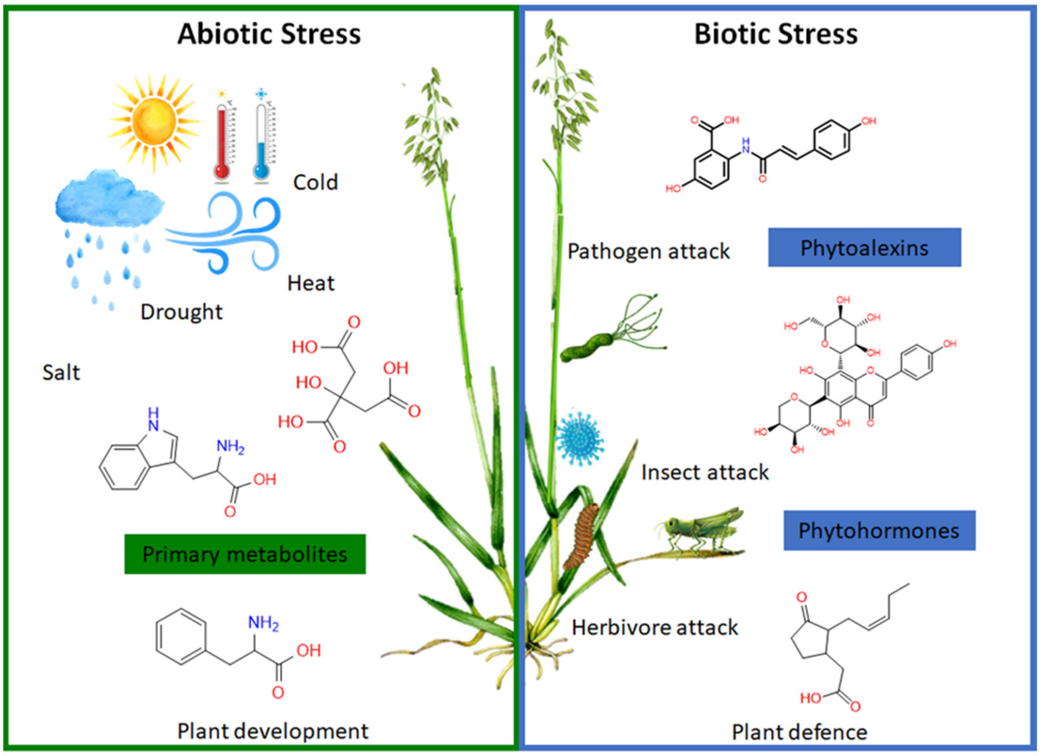

Figure S1. Key metabolic components that underlie plant responses to abiotic and biotic stressors. Plant metabolomics is a powerful tool for studying the metabolic mechanisms that trigger and embody plant stress responses to environmental (left) and/or pathogenic (right) threats.

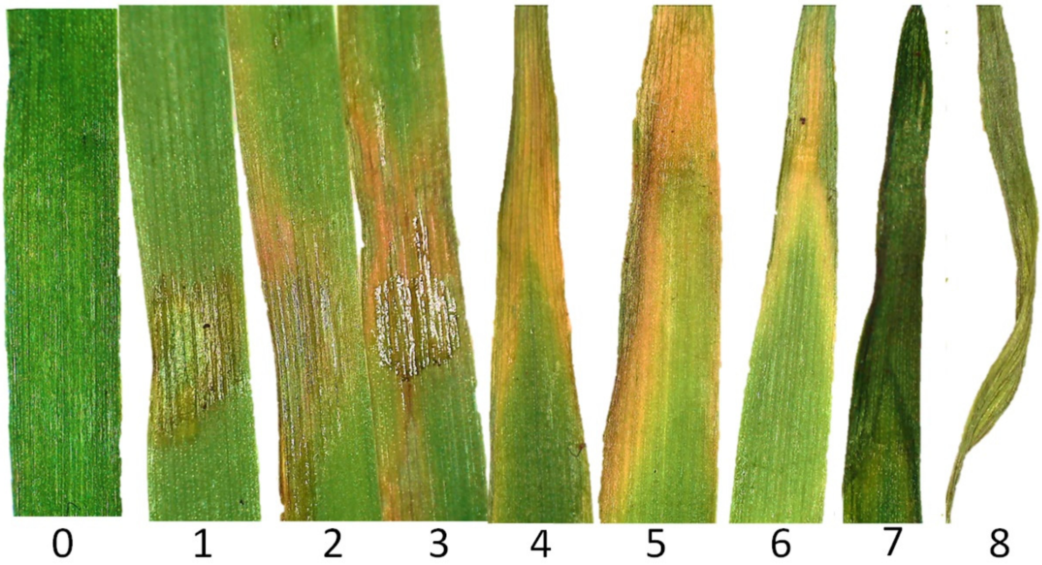

Figure S2. Halo blight disease severity index on *Avena sativa* L. inoculated with *Pseudomonas syringae* pv. *coronafaciens*. Example images represent visual observation of disease severity using a 0 - 8 scale, where 0 = no disease symptoms, 1-3 = slight disease symptoms, 3-6 = moderate disease symptoms and 6-8 = severe symptoms of yellowing and wilting.

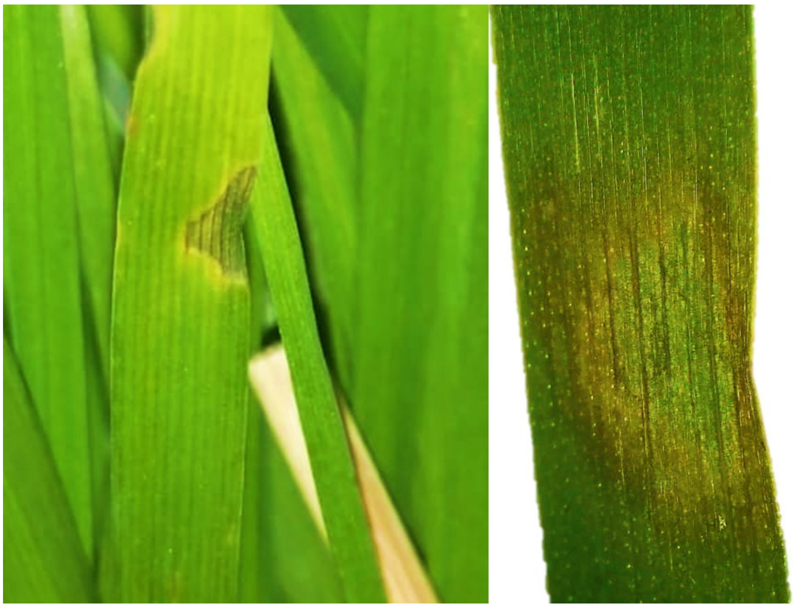

Figure S3. Typical symptom development of halo blight on 'Dunnart' leaves in response to *Ps-c* infection. Characteristic yellow halo formation around infected leaf tissue, typically associated with halo blight disease.

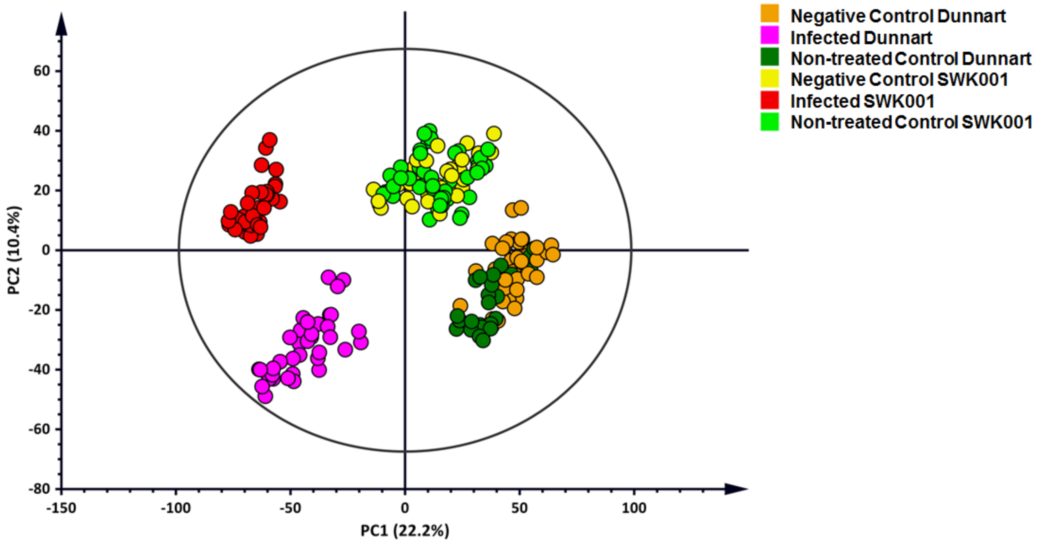

Figure S4. Principal component analysis (PCA) of the ESI(−) data illustrating the two infected oat cultivars and the respective control groups. The PCA scores plot of all the samples showing the infected and control groups (non-treated vehicle- and negative control) for 'Dunnart' and 'SWK001'. The non-treated and negative controls clustered together for both cultivars, meaning that there were no significant differences among the controls, i.e. the underlying metabolic profiles are similar compared to the infected groups.

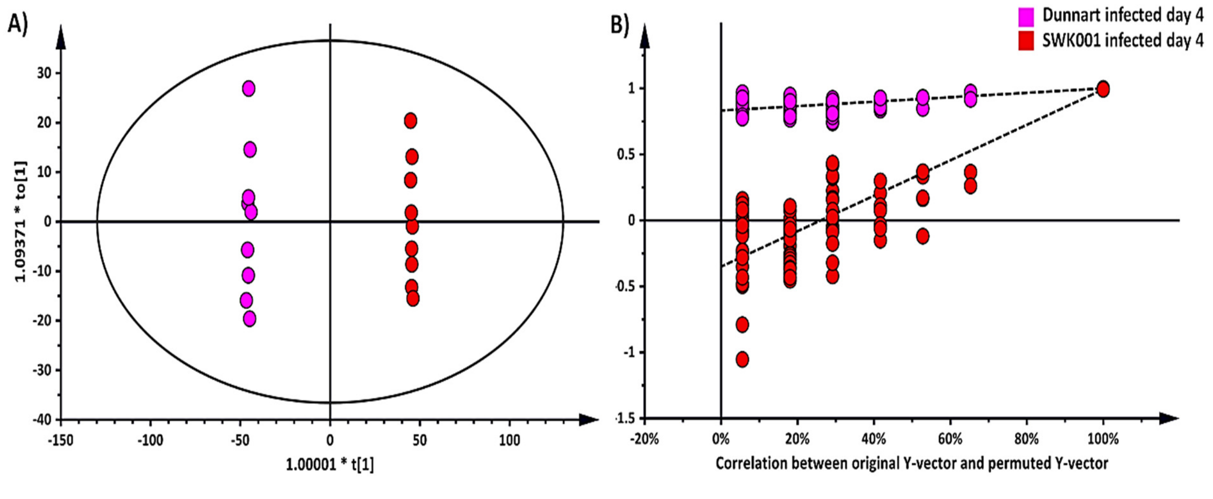

Figure S5. An orthogonal projection to latent structures discriminant analysis (OPLS-DA) model of the two infected cultivars, **'**Dunnart' and 'SWK001'. **(A)** OPLS-DA scores plot illustrating the relationship between the two cultivars at 4 d.p.i. based on their leaf-extracted metabolic profiles obtained in ESI (–) mode (R^2=0.999, Q^2=0.996, CV-ANOVA *p*-value= 1.06365×10^{-14}). **(B)** Permutation test plot (*n* = 100) for the OPLS-DA model **(A)** was used to validate the predictive capability.

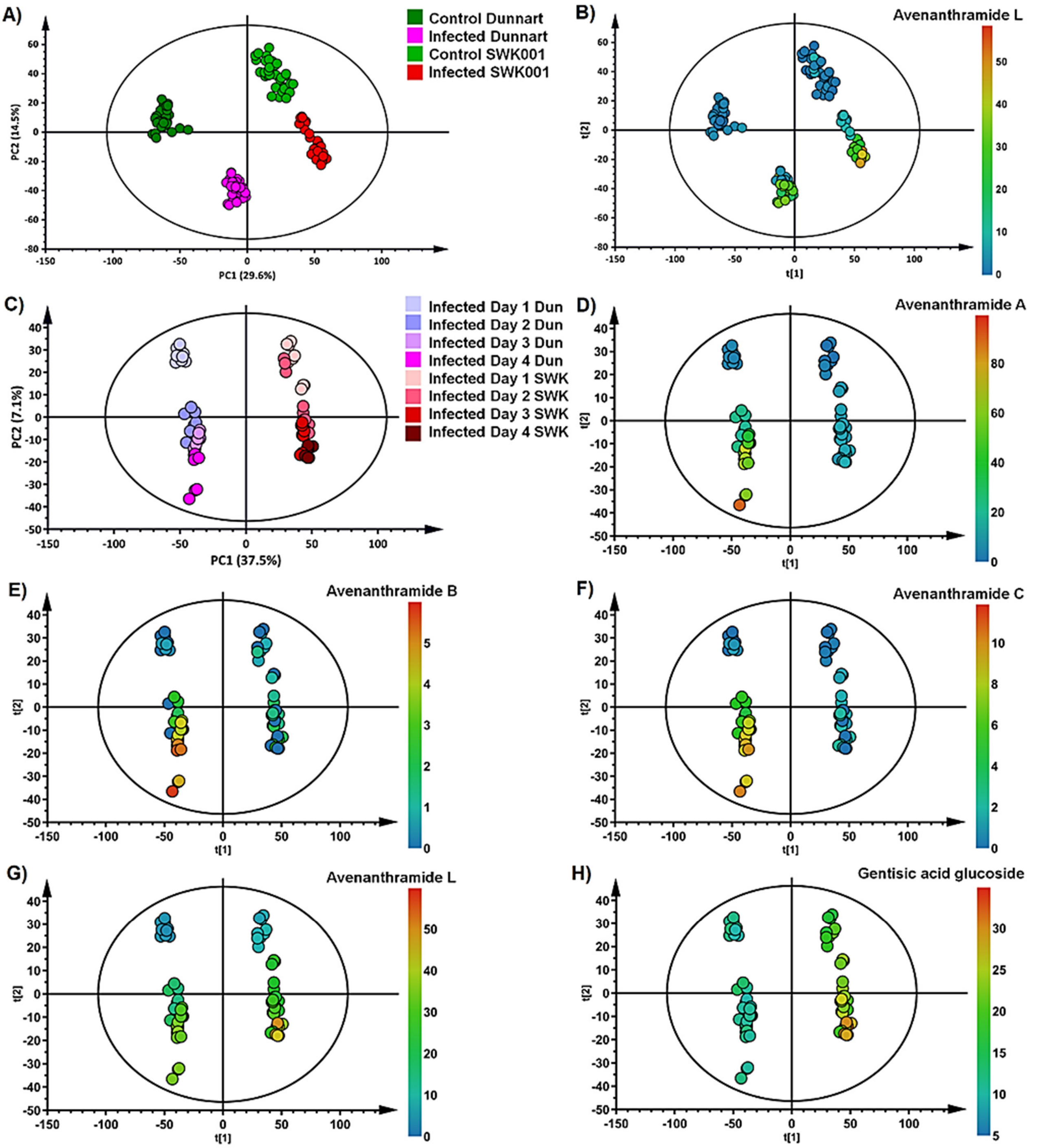

Figure S6. Colour coded PCA score plots showing the presence and increasing abundance of discriminatory ions in the respective treated cultivars (ESI− mode), 'Dunnart' & 'SWK001', (red- high abundance, blue- low abundance). **(A)** PCA scores plot of both cultivars showing control and infected groups, **(B)** Avenanthramide L (absent in control groups and present in infected groups), **(C)** PCA scores plot showing the infected groups for both cultivars from 1 d.p.i. to 4 d.p.i. **(D)** Avenanthramide A, **(E)** Avenanthramide B, **(F)** Avenanthramide C, **(G)** Avenanthramide L and **(H)** Gentisic acid glucoside.

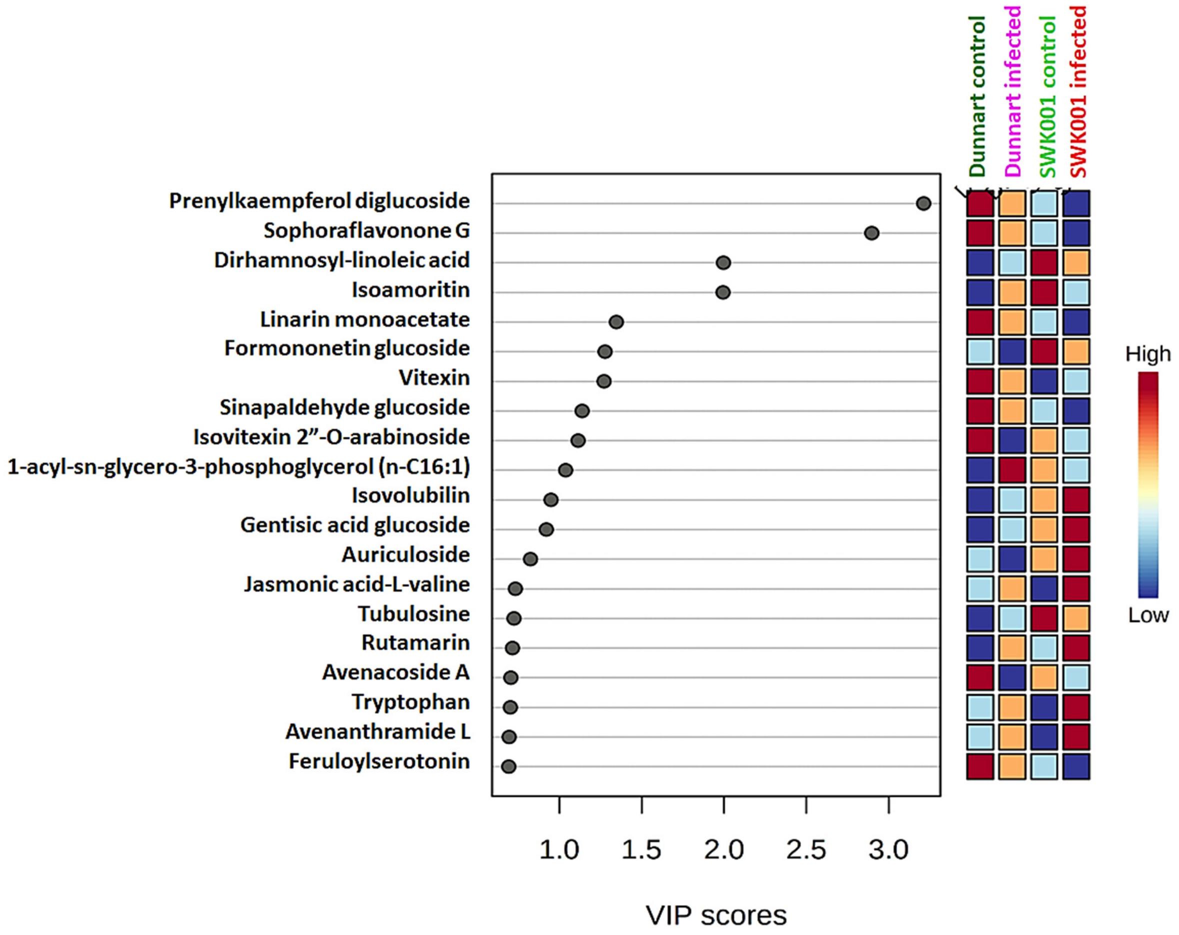

Figure S7. Variable importance in projection (VIP) scores generated using MetaboAnalyst software. Indicated are the top 15 discriminating ions in oat leaves from the respective cultivars ‘Dunnart’ & ‘SWK001’ for their treated and control groups. Metabolites with a VIP score ≥0.5 were considered to be significant in the discrimination between the cultivars.

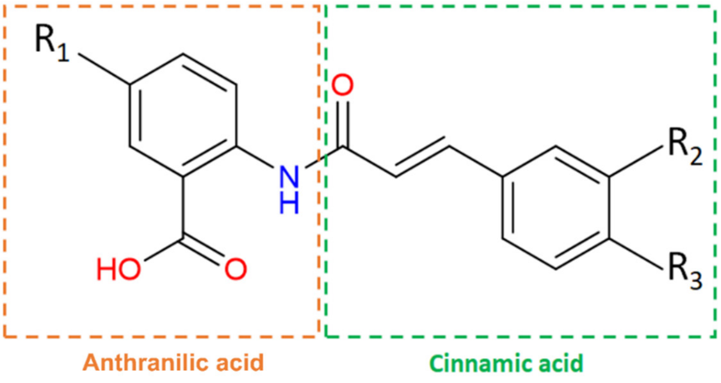

Compound	R_1	R_2	R_3	**Anthranilic acid**	**Cinnamic acid**
Avenanthramide A	OH	H	OH	5-hydroxyanthranilic acid	*p*-coumaric acid
Avenanthramide B	OH	OCH_3	OH	5-hydroxyanthranilic acid	ferrulic acid
Avenanthramide C	OH	OH	OH	5-hydroxyanthranilic acid	caffeic acid

Figure S8. Avenanthramide structures illustrating the core structure and various functional groups unique to each compound.

***Pseudomonas syringae* pv. *coronafaciens* 16S rRNA sequence (Inqaba)**

GTCACTTAATGCGTTAGCTGCGCCACTAAGAGCTCAAGGCTCCCAACGGCTAGTTGACAT

CGTTTACGGCGTGGACTACCAGGGTATCTAATCCTGTTTGCTCCCCACGCTTTCGCACCT

CAGTGTCAGTATCAGTCCAGGTGGTCGCCTTCGCCACTGGTGTTCCTTCCTATATCTACG

CATTTCACCGCTACACAGGAAATTCCACCACCCTCTACCATACTCTAGCTTGCCAGTTTT

GGATGCAGTTCCCAGGTTGAGCCCGGGGATTTCACATYCAACTTAACAAACCACCTACGC

GCGCTTTACGCCCAGTAATTCCGATTAACGCTTGCACCCTCTGTATTACCGCGGCTGCTG

GCACAGAGTTAGCCGGTGCTTATTCTGTCGGTAACGTCAAAACAATCACGTATTAGGTAA

CTGCCCTTCCTCCCAACTTAAAGTGCTTTACAATCCGAAGACCTTCTTCACACACGCGGC

ATGGCTGGATCAGGCTTTCGCCCATTGTCCAATATTCCCCACTGCTGCCTCCCGTAGGAG

TCTGGACCGTGTCTCAGTTCCAGTGTGACTGATCATCCTCTCAGACCAGTTACGGATCGT

CGCCTTGGTGAGCCATTACCTCACCAACTAGCTAATCCGACCTAGGCTCATCTGATAGCG

CAAGGCCCGAAGGTCCCCTGCTTTCTCCCGTAGGACGTATGCGGTATTAGCGTCCGTTTC

CGAGCGTTATCCCCCACTACCAGGCAGATTCCTAGGCATTACTCACCCGTCCGCCGCTCG

CCACCAGGTACAAGTACCCGTGCTGCCGCTCGACTTGCATGTGTTAGGCCTGCCGCCAGC

GTTCAATCTGAGCCATGATCAAAACWCTGAAG

Figure S9. 16S rRNA sequence of *Pseudomonas syringae* pv. *coronafaciens*. The sequence exhibits 99.19 % identity with that of *P. coronafaciens* X-1, causing halo blight disease in oats [19].
